# Optimizing Imaging Parameters for Assessment of Hepatocellular Carcinoma Using Photon-Counting Detector Computed Tomography—Impact of Reconstruction Kernel and Slice Thickness

**DOI:** 10.3390/tomography11070077

**Published:** 2025-06-27

**Authors:** Anna Szelenyi, Philipp Stelzer, Christian Wassipaul, Jakob Kittinger, Andreas Strassl, Victor Schmidbauer, Martin Luther Watzenböck, Florian Lindenlaub, Michael Arnoldner, Michael Weber, Matthias Pinter, Ruxandra-Iulia Milos, Dietmar Tamandl

**Affiliations:** 1 Department of Biomedical Imaging and Image-Guided Therapy, Medical University of Vienna, 1090 Vienna, Austria; 2 Division of Gastroenterology and Hepatology, Medical University of Vienna, 1090 Vienna, Austria

**Keywords:** photon counting detector computed tomography, hepatocellular carcinoma, kernel reconstructions, spatial resolution

## Abstract

Background: The use of photon-counting detector computed tomography (PCD-CT) has improved image quality in cardiac, pulmonary, and musculoskeletal imaging. Abdominal imaging research, especially about the use of PCD-CT in hepatocellular carcinoma (HCC), is sparse. Objectives: We aimed to compare the image quality of tumors, the liver parenchyma, and the vasculature in patients with HCC using PCD-CT reconstructions at different slice thicknesses and kernels to identify the most appropriate settings for the clinical routine. Methods: CT exams from twenty adult patients with HCC performed with a clinically approved, first-generation PCD-CT scanner (Naeotom Alpha^®^, Siemens Healthineers), were retrospectively reviewed. For each patient, images were reconstructed at four different sharp kernels, designed for abdominal imaging (Br40; Br44; Br48; Br56) and at three slice thicknesses (0.4 mm; 1 mm; 3 mm). The reconstruction with the Br40 kernel at 3 mm (Br40_3 mm_) was used as a clinical reference. Three readers independently assessed the image quality of different anatomical abdominal structures and hypervascular HCC lesions using a five-point Likert scale. In addition, image sharpness was assessed using line-density profiles. Results: Compared with the clinical reference, the Br44_1 mm_ and Br48_1 mm_ reconstructions were rated superior for the assessment of the hepatic vasculature (median difference +0.67 [+0.33 to +1.33], *p* < 0.001 and +1.00 [+0.67 to +1.67], *p* < 0.001). Reconstructions for Br40_1 mm_ (+0.33 [−0.67 to +1.00], *p* < 0.001), and Br44_3 mm_ (+0.0 [0.0 to +1.00], *p* = 0.030) were scored superior for overall image quality. The noise demonstrated a continuous increase when using sharper kernels and thinner slices than Br40_3 mm_ (*p* < 0.001), leading to a decrease in contrast-to-noise ratio. Although there was a trend toward increased image sharpness using the slope analysis with higher kernels, this was not significantly different compared with the reference standard. Conclusion: PCD-CT reconstruction Br40_1 mm_ was the most suitable setting for overall image quality, while reconstructions with sharper kernels (Br44_1 mm_ and Br48_1 mm_) can be considered for the assessment of the hepatic vasculature in patients with HCC.

## 1. Introduction

Photon-counting detector computed tomography (PCD-CT) is an innovative image acquisition concept. Compared with energy-integrating detector CTs (EID-CT), it has been shown to improve resolution and reduce image noise and thus potentially improve image quality in cardiothoracic, musculoskeletal, and abdominal imaging [1,2,3,4,5]. Compared with conventional EID-CT-scanners, the key technical advance of PCD-CT is the ability to measure energies of incident photons that hit the semiconducting detector and convert it straight into a proportional electrical signal, thus enabling material decomposition and reducing electronic noise [6,7]. Furthermore, the geometric dose efficiency and spatial resolution are improved when compared with the standard acquisition method [6,8]. Taken together, these promising advantages of PCD-CT have been shown to decrease radiation dose [9], reduce noise [10,11], improve contrast-to-noise ratio [6,11,12,13], and increase spatial resolution [10] when compared with EID-CT [9,13,14,15].

Due to the improved resolution capabilities of PCD-CT, the currently recommended slice thickness and reconstruction kernels for EID-CT might not provide the optimal image quality for PCD-CT. Adjustments to protocols for EID-CT have already been described for cardiac and lung imaging [14,16], as well as for some abdominal imaging indications [17]. A reconstruction kernel is a mathematical function applied during image reconstruction to emphasize or suppress certain spatial frequencies in the raw scan data. The choice of kernel significantly influences the final image appearance: sharp kernels enhance edges and fine structures but increase noise, while smooth kernels reduce noise but may obscure detail. Therefore, kernel selection is a critical factor in optimizing the balance between noise and resolution in CT imaging. Given the different image characteristics of PCD-CT compared with EID-CT, a new standardization of reconstruction kernels and slice thickness for abdominal examinations is necessary.

Hepatocellular carcinoma (HCC) is the most frequent primary liver tumor in the setting of liver cirrhosis [18] and requires a dedicated imaging protocol not only for primary diagnosis but also for follow-up imaging [19]. This is related not only to enhanced visualization of the arterial vasculature for liver-directed interventions, but also the challenging feature of increased background noise in liver cirrhosis.

Several studies have compared the image quality between PCD-CT and EID-CT [4,12,20,21], but detailed information about reconstruction parameters in abdominal imaging, and especially in HCC imaging in cirrhotic patients, is lacking.

Schwartz et al. [17] evaluated abdominal imaging protocols across various institutions, emphasizing the absence of a unified consensus on the optimal approach. Sartoretti et al. [22] concentrated on enhancing image quality in abdominal imaging by exploring the most efficient quantum iterative reconstruction method. Graafen et al. [4] showcased superior image quality in PCD-CT compared with EID-CT through the application of low-keV virtual monoenergetic images. However, no studies, to date, have directly compared images acquired at different slice thicknesses and reconstruction kernels within the same study for HCC.

Therefore, the aim of this exploratory study was to assess the optimal image quality in patients undergoing routine staging of HCC using the first-generation PCD-CT by assessing different slice thickness and different sharp reconstruction kernels.

## 2. Materials and Methods

### 2.1. Patient Recruitment

Between February and November 2022, 47 consecutive patients who underwent a clinically indicated abdominal PCD-CT for the staging or restaging of HCC, including an arterial, portal venous, and delayed phase, were potentially eligible for this study. Seven patients with treated and thus non-hypervascular liver lesions were excluded, and seventeen patients did not exhibit any HCC liver lesions in the setting of a follow-up study after treatment. Three patients had to be excluded because of missing image reconstructions. Clinical parameters were retrieved from the hospital information system. This retrospective study was approved by the local institutional review board (Nr.: 2032/2021), and written, informed consent was waived. Data were pseudonymized and all readings were performed without revealing the patients’ data to the readers.

### 2.2. Image Acquisition

All CT examinations were acquired using a first-generation PCD-CT scanner (Naeotom Alpha^®^, Siemens Healthineers, Erlangen, Germany), with a routine clinical HCC protocol, which included a standardized, weight-based contrast injection protocol (IOMERON 400, Bracco Inc., Milan, Italy). Table 1 shows the dose length product (DLP) and dose indices (CTDIvol), as well as contrast agent volumes, that were recorded.

### 2.3. Image Reconstruction

For each patient, the same raw data taken from the arterial phase were reconstructed in 12 data sets of images using four different sharpness kernels (Br40, Br44, Br48, and Br56) and three different slice thicknesses of 0.4 mm, 1 mm, and 3 mm. A polyenergetic dataset was acquired at 120 kVp to ensure high-quality, low-noise data with excellent tissue penetration. Level 3 quantum iterative reconstruction was used for image reconstruction. Images were reconstructed at 60 kilo-electronvolts to optimize contrast and enhance diagnostic utility for abdominal imaging. The median pixel size was 0.7 mm (0.62 mm–0.88 mm) with a variable field of view from 317 mm to 451 mm (median 358 mm) and a fixed matrix of 512 × 512. An average volume of 77 ± 8 mL of contrast agent was injected at an average flow rate of 3.5 ± 0.5 mL/s. Acquisition and reconstruction details are also displayed in Table 1.

### 2.4. Qualitative Image Analysis

A radiology technologist pseudonymized the image reconstructions and stored them separately on a clinical viewing workstation, with the Picture Archive and Communications System (PACS). For subjective image quality analysis, the pseudonymized images of each patient were independently loaded and displayed using the PACS, ensuring that each image was assessed on its own. The reconstruction using the Br40 kernel and a 3 mm slice thickness, which is the vendor-recommended setting, served as the clinical reference. Each image was evaluated individually against this reference, without side-by-side comparison of different reconstructions. For image quality assessment, three radiologists (two to four years of experience), blinded to the PCD-CT reconstruction method, independently evaluated the randomly assigned images in relation to the reference standard. For each review, the readers were asked to assess their ability to rate overall image quality; conspicuity; sharpness of liver masses; intra- and extrahepatic biliary tract; lymph nodes; falciform ligamentum; renal cortex; and arterial vessels (hepatic artery, superior mesenteric artery, and aorta) compared with the reference standard. The readers were asked to identify the highest-level hepatic artery branch clearly identifiable in the liver. The common hepatic artery (CHA) was considered to be of the first order. For multifocal HCC, the readers were asked to evaluate the most suitable lesion, identified as either the largest or the most prominently enhancing lesion.

Rating was performed according to a five-point Likert scale from −2 to +2:

−2 = definitely worse, definitely reduced diagnostic ability

−1 = worse, unclear effect on potential diagnosis

0 = about the same or unclear benefit/decrement

+1 = better, unclear effect on potential diagnosis

+2 = definitely better, increased diagnostic ability

### 2.5. Quantitative Analysis

A non-reader radiologist measured CT numbers in various abdominal structures to obtain objective noise and contrast-to-noise ratio of the liver parenchyma, aorta, liver lesions, renal cortex, the common hepatic artery, and the iliopsoas muscles. Fixed circular regions of interest (ROIs) were placed while avoiding vessels in the liver parenchyma, and any opacities at the level of the liver hilum. An ROI of 1 cm in diameter was chosen, if possible, and then copied to all reconstruction kernels. For arterial vessels smaller than 1 cm, the largest possible ROI was drawn. Image noise was measured using the standard deviation of CT numbers in the psoas muscle. Contrast-to-noise ratio was calculated as CNRstructure = (mean structure attenuation–liver attenuation)/noise.

Furthermore, another non-reader radiologist measured objective sharpness of the kidney parenchyma using line-density profiles. A 1 cm line was drawn, which covered the renal cortex, medulla, and adjacent fatty tissue. Maximum and minimum HU values, as well as the distance between those values, were recorded and the slope (increase or decrease) was calculated using ImageJ (Version 1.54j), as previously reported [22]. For the purpose of this analysis, a representative slice with homogeneous kidney parenchyma and surrounding fat was chosen. Caution was undertaken, not to measure into any lesions or adjacent structures.

### 2.6. Statistical Analysis

The values of the image quality and the secondary objectives (conspicuity and sharpness of the intra-, extrahepatic biliary tract, lymph nodes, vessels, falciform ligament, renal cortex, and liver pathology) were reported as median and range. The results of subjective image quality assessment were ascertained for each reader separately and also as a pooled analysis to reduce the influence of the subjectivity of a single rater. For a simple comparison of the results of the image quality, a scoring model was used to assign a comparative value for each kernel and structure. The outcomes for all 20 patients were added together for all three readers separately (e.g., if a reader assessed the arterial vascularity for the reconstruction Br44_1 mm_ in all twenty patients with 1, then the summed score would be 20) and aggregated for a clear representation. Three-dimensional stacked column charts were used for a graphical representation of the data.

Likewise, for the mere descriptive evaluation of the CNR and for image sharpness, the average values +/− standard deviation (in the case of the normal distribution and the median and quartile for the non-normal distribution) were calculated for each set and image reconstruction separately. Boxplots and heatmaps were used for graphic representation.

Variables were assessed for distribution using the Kolmogorov–Smirnov test. Two-sided *t*-tests for normally distributed data and the Wilcoxon test for paired, non-parametric data, where applicable, were used to compare continuous variables. The Chi-square test was used to compare categorical data. Unifactorial analysis of variance with Bonferroni correction was used for the slope analysis. Two-way repeated measure ANOVAs were used to compare CNR measures for different kernels and slice thicknesses. To compare ordinal data, generalized estimation equations were used to take repeated measures per patient into account. Inter-reader agreement among the three readers was assessed using κ coefficients.

*p*-values below 0.05 were considered statistically significant. To avoid an increased error of the second type, no multiplicity corrections were performed.

## 3. Results

### 3.1. Patients and Demographic Details

A total of 20 patients (19 male and one female, median age 62.5 years; range 39–85 years, median BMI 25; range 20–29.6) with known HCC lesions and liver cirrhosis were included in the final study cohort. Most HCC lesions were multifocal (17 of 20 patients) with a median size of the largest lesion in each patient of 3.1 cm (range 1.2–13.7 cm) in diameter. Vascular invasion of the HCC lesions was detectable in the CT examination in six patients. Furthermore, one patient had lung metastases, one patient had peritoneal secondary lesions, and one patient had adrenal and bone metastases.

### 3.2. Objective Image Quality

Noise demonstrated a continuous increase when using sharper kernels and thinner slices (*p* < 0.001 in the two-way ANOVA analysis). The least noise was perceived at Br40_3 mm_, and the most at Br56_0.4 mm_, as per Table 2. Consequently, CNR decreased in all slice thicknesses compared with the reference standard for each assessed property (e.g., CNR of liver lesion 4.5 ± 2.0 vs. 3.0 ± 1.2 vs. 2.0 ± 0.7 for Br40_3 mm_, Br40_1 mm,_ and Br40_0.4 mm_, respectively, *p* < 0.001). CNR was also highest for the softest kernel, as expected (e.g., 4.5 ± 2.0 vs. 3.5 ± 1.5 vs. 2.7 ± 1.2 vs. 2.0 ± 0.8 for Br40 vs. Br44 vs. Br48 vs. Br56, respectively, Table 2, *p* < 0.001).

### 3.3. Subjective Image Quality

**Figure 1:**
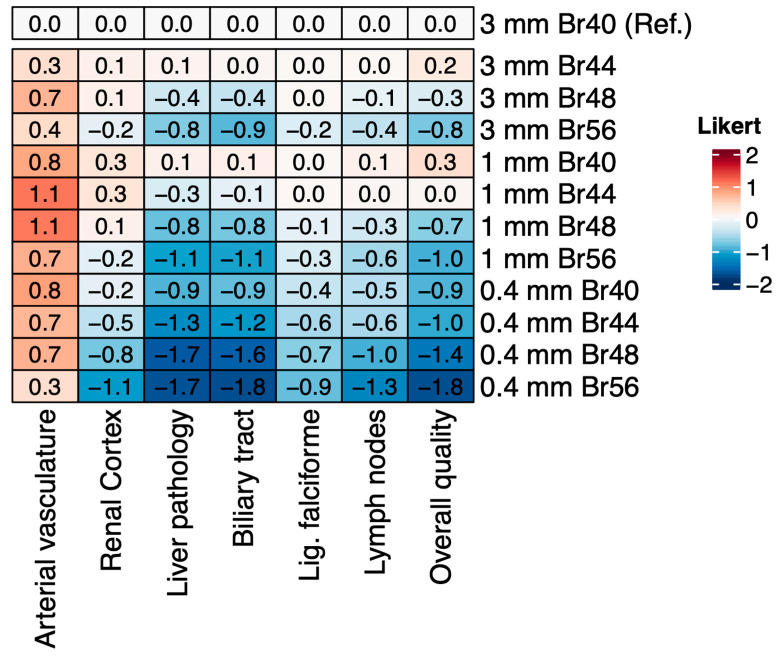
Heatmap of qualitative image assessment. For each structure, Likert scores of all readers were pooled and color coded. Red indicates better image quality and blue denotes inferior image quality, the Likert scale ranged from −2 (definitely worse) to +2 (definitely better), with 0 indicating no change from the reference. Br40_3 mm_ was chosen as the reference standard.

**Figure 2:**
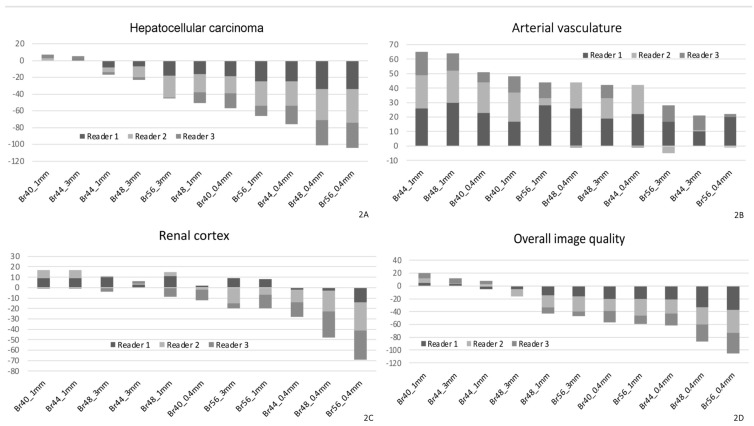
Pooled Likert scores for each structure, assessed and pooled for each reader. For the purpose of this analysis, each Likert rating for each analyzed reconstruction setting was added or subtracted according to the score (e.g., three individual readings of +2, +1, and −2 would result in +1). Results are shown for liver pathology (HCC, (**A**)), the arterial vasculature (**B**), the renal cortex (**C**), and overall image quality (**D**). Br40_3 mm_ served as the reference standard to which the Likert score was compared, which is identical to the position of the x-axis. The x-axis denotes the reconstruction set with kernel and slice thickness settings; the y-axis denotes the sum of all Likert scores for that respective set.

**Figure 3:**
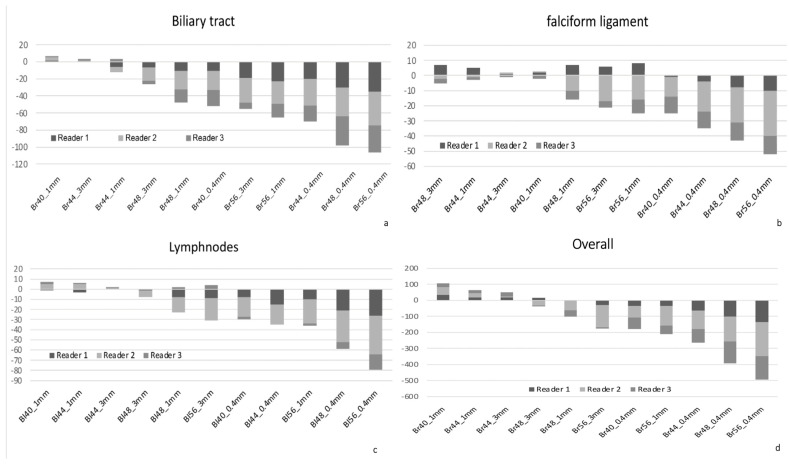
Additional structures assessed with the pooled Likert score analysis, similar to Figure 2. For the purpose of this analysis, each Likert rating for each analyzed reconstruction setting was added or subtracted according to the score (e.g., three readings of +2, +1, and −2 would result in +1). Results are shown for the biliary tract (**a**), the falciform ligament as a surrogate for structures adjacent to the liver (HCC, (**b**)), lymph nodes (**c**), and pooled overall assessments for all structures (**d**). Br40_3 mm_ served as the reference standard to which the Likert score was compared. The x-axis denotes the reconstruction set with kernel and slice thickness settings; the y-axis denotes the sum of all Likert scores for that respective set.

The pooled Likert scores of all three readers for each assessed structure are displayed in a heatmap in Figure 1, with the distribution of summed ratings visualized for all three readers separately in Figure 2A–D and Figure 3a–d.

In the evaluation of HCC lesions, only Br40_1 mm_ (median difference 0.0 [−0.33 to +0.67], *p* = 0.013] showed minor improvements, while Br44_3 mm_ was rated similarly (0.0 [0.0 to +1.00], *p* = 0.468), whereas all other reconstructions were rated inferior to the reference standard (e.g., Br44_1 mm_ −0.33 [−1.00 to +0.33], *p* < 0.001, Figure 3a).

The largest subjective improvement in image quality was observed for vascular structures at 1 mm slice thickness, with kernels Br44_1 mm_ and Br48_1 mm_ performing best vs. the reference standard (median difference [range] +0.67 [+0.33 to +1.33], *p* < 0.001 and +1.00 [+0.67 to +1.67], *p* < 0.001, respectively, Figure 1 and Figure 2B). There was a minor improvement in Br40_1 mm_ over the reference standard for the assessment of the renal cortex (+0.17 [+0.00 to +0.67], *p* = 0.006, Figure 1 and Figure 2), which was, in general, more equivocally rated between readers, with higher interreader disagreement compared with other structures. In the sum of the assessments for the renal cortex, the Br40_1 mm_ and Br44_1 mm_ reconstructions were rated best, similar to the sharper kernels Br48_3 mm_ and Br 48_1 mm_, respectively, although the difference was only minimally better compared with the reference standard.

Overall image quality was perceived best at Br40_1 mm_ (+0.33 [−0.67 to +1.00], *p* < 0.001), and Br44_3 mm_ (+0.0 [0.0 to +1.00], *p* = 0.030), while Br44_1 mm_, although numerically higher, was not rated significantly better (+0.17 [−1.00 to +1.00], *p* = 0.366), as per Figure 2D. Additional results are summarized in Figure 3. In general, reconstructions at 0.4 mm slice thickness and kernels higher than Br44 were rated inferior for all instances, except vasculature. Inter-reader variability was fair to moderate (Cohen’s κ range 0.276−0.482).

### 3.4. Assessment of Image Sharpness

**Figure 4:**
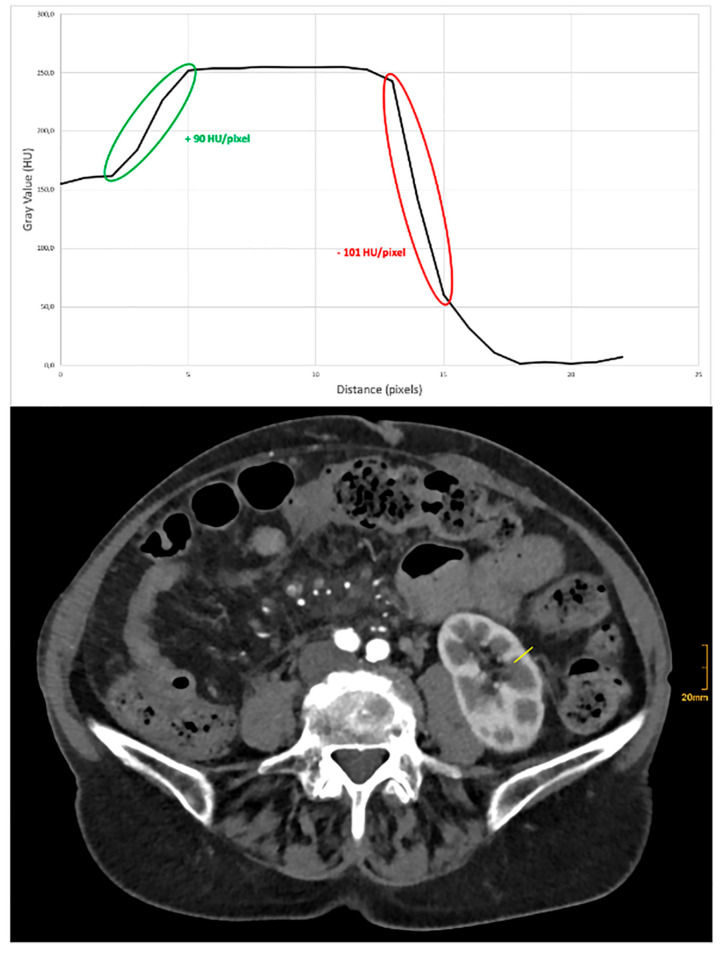
Example of a line density profile for objective analysis of image sharpness. The ascending (green) part of the slope refers to the change of density between renal medulla and cortex, while the descending (red) slope refers to the density change between renal cortex and perirenal fat. Steeper curves correlate with a higher degree of image sharpness.

Image sharpness, expressed as the steepness of the CT number gradient of the renal medulla:renal cortex and renal cortex:renal fat, respectively, was higher in the group with the thinner slice thickness (mean HU [HU/mm] for 0.4 mm 39.1 ± 12.7 vs. 31.2 ± 9.0 vs. 35.6 ± 9.6 for 1.0 mm and 3.0 mm, respectively, for medulla:cortex, and −86.0 ± 29.9 vs. −75.9 ± 22.5 vs. −80.6 ± 22.6, respectively, for cortex:fat). The impact of various kernels on image sharpness was less pronounced (mean HU Br40 vs. Br44 vs. Br 48 vs. Br56: 35.8 ± 11.8 vs. 34.8 ± 10.8 vs. 33.9 ± 10.6 vs. 36.7 ± 10.9, respectively, for medulla:cortex and −79.6 ± 24.4 vs. −84.3 ± 23.2 vs. −82.3 ± 32.5 vs. −77.0 ± 20.6, respectively, for cortex:fat). Regardless, there was no statistical difference between either variations of slice thicknesses or kernels (*p* > 0.05). For an example of a line density profile, see below Figure 4.

### 3.5. Assessment of Intrahepatic Vasculature

The highest intrahepatic dichotomization that was visible on the reference standard was 11. Minor deviations were depicted in different reconstructions, with Br44_1 mm_ and Br48_1 mm_ scoring best in median (6) and in highest intrahepatic dichotomization (12). However, interreader agreement was poor (range of dichotomization from 3 to 12, Cohen’s κ < 0.3), thus limiting the practical relevance of this feature.

## 4. Discussion

In this study, image quality and optimal reconstruction parameters for the imaging of hepatocellular carcinoma, the hepatic vasculature, and other upper abdominal structures were assessed for PCD-CT. This study demonstrated that certain combinations of reconstruction kernel and slice thickness led to superior subjective image quality compared with the reference standard, especially for the hepatic vasculature, renal cortex assessment, and overall image quality. However, most of the tested reconstructions, especially with higher kernels beyond Br44 and slice thicknesses thinner than 1 mm (except vascular imaging), showed a slight to clear disadvantage in the image assessment.

Higher kernels are generally considered sharper and are typically more suitable for imaging high-contrast structures such as bones and lungs. However, in the context of PCD-CT, the new detector technology appears to influence the optimal reconstruction settings for delicate structures like blood vessels. The observed modest improvement with higher kernels (e.g., Br44 and Br48 at 1 mm slice thickness) for vascular assessment could be due to the enhanced edge definition and contrast resolution provided by the photon-counting detector. This technology improves the visualization of fine details while maintaining noise at a manageable level, making sharper kernels more beneficial for vascular imaging.

Conversely, for the assessment of the abdominal parenchyma and HCC, Br40 at 1 mm slice thickness demonstrated the best overall performance. This suggests that further increasing the kernel sharpness and reducing the slice thickness did not provide additional diagnostic value. Instead, it may have introduced more image noise or compromised soft-tissue contrast, which is crucial for the evaluation of parenchymal structures. The fact that Br40_1 mm_ and Br44_3 mm_ yielded the best image quality overall indicates that a balance between spatial resolution, noise, and soft-tissue contrast is necessary, with the improved energy resolution of PCD-CT playing a key role in optimizing these parameters.

Since the technological advance of photon-counting CT, with promised improvements in spatial resolution, contrast-to-noise ratio, dose efficiency, and spectral imaging, most studies have focused on the evaluation of these benefits compared with the standard, which, so far, has been EID-CT [4,10,11,12,13,23]. Although various groups, e.g., Schwartz et al. and Graafen et al. [4,23], have emphasized the importance of standardized acquisition parameters in abdominal imaging, no studies, to date, have compared images in patients at different slice thicknesses and kernels within the same study in HCC. The assessment of HCC is significantly more challenging in patients with liver cirrhosis due to altered parenchymal texture, fibrosis, and potential perfusion abnormalities. Based on the findings of this study, the default setup of Br40_3 mm_ appears to be a safe and well-balanced choice for the evaluation of a broad range of abdominal structures. However, Br40_1 mm_ demonstrated superior performance specifically for the assessment of the abdominal parenchyma and HCC. In clinical implementation, this suggests that Br40_1 mm_ should be preferred in cases where lesion conspicuity and contrast resolution are critical, such as in patients with liver cirrhosis and HCC, where assessment is significantly more challenging.

**Figure 5:**
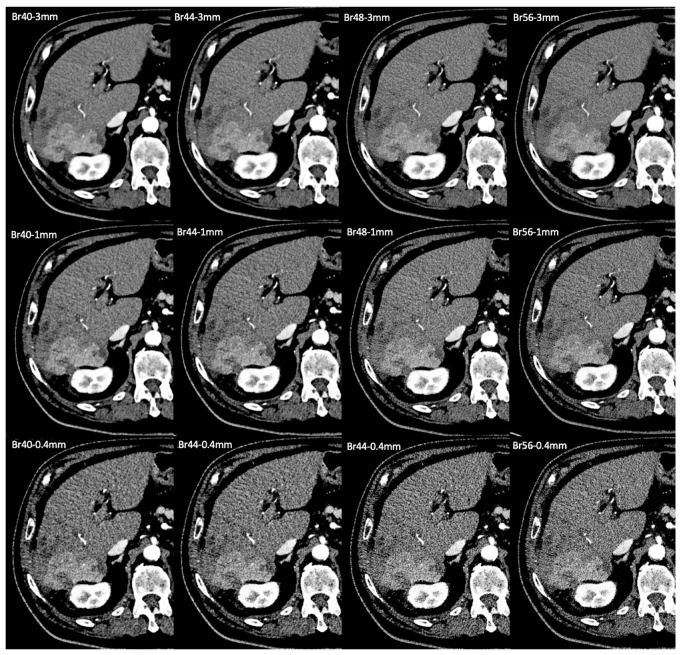
Examples of photon-counting CT in the arterial phase in a patient with liver cirrhosis and a hepatocelluar carcinoma (HCC) in the right posterolateral sector. Each subimage represents a certain reconstruction setting, which is depicted in the left upper corner of the image. Br40_3 mm_, which served as the reference standard, was compared with each set. One mm reconstructions were rated higher for vascular assessment (best for kernels Br 44 and Br 48) and slightly better for overall image quality in the Br40 kernel. Although noise was lowest in the standard setting, readers felt the increased sharpness of higher kernels and thinner slices was beneficial for the assessment of smaller vessels and structures with a clear edge, like the renal cortex.

In our study, the standard kernel and slice thickness was, overall, a good mix for the assessment of HCC and other upper abdominal structures, with only minor improvement with Br44 and Br48 in single instances. The improvement of vascular imaging at 1 mm slice thickness with kernels Br44 or Br48 warrants further research to determine whether this type of reconstruction should be separately performed, especially if interventional procedures, such as transarterial chemoembolization, are to be planned. However, the submillimeter slice thickness of 0.4 mm and the very sharp kernels of Br56 did not bring any significant advantage for vessel assessment. An example of all of the reconstruction sets is given in Figure 5. Similar to what was described by Milos et al. [16] for thoracic imaging, using a submillimeter slice thickness (0.4 mm) did not result in any additional value for the visualization of other abdominal structures or liver pathology. Instead, this only led to worse image quality. On the other hand, Mergen et al. have demonstrated a decreased vessel sharpness and more blooming artifacts, but better CNR, SNR, and lower image noise when using 0.6 mm slice thickness compared with 0.2 mm for ultra-high-resolution cardiac imaging [14]. Similarly, lower kernel sharpness resulted in decreased vessel sharpness and more blooming artifacts. However, this study was performed in the ultra-high-resolution mode that was not available in our patients.

This study has several limitations. This was a single-center pilot study, and, while in the size range of similar studies [4,24,25,26], only a limited number of patients was evaluated. While the observed trends were consistent and statistically significant in several relevant aspects, larger multi-center studies with more heterogeneous patient populations are warranted to validate and refine our observations. Such studies would further strengthen the clinical relevance and applicability of the optimized reconstruction settings proposed here. The purpose of this pilot study was to optimize our routine protocol and to guide further research regarding potential clinical applications of PCD-CT for HCC imaging.

A comparison of PCD-CT to EID-CT at similar slice thicknesses and comparable kernels, as well as an evaluation of abdominal structures in the ultra-high-resolution mode, was not performed. This is a possible direction for future studies in this field. In addition, the results of this study are specific to the current Siemens photon-counting CT system and may not be directly applicable to other vendors or future hardware generations.

We acknowledge that inter-reader agreement in our study was only fair to moderate (Cohen’s Kappa 0.276–0.482) for the qualitative assessments. This level of variability is a common limitation in studies based on subjective image quality evaluation and reflects individual differences in reader perception and emphasis on specific image features. In our study, three readers with varying levels of experience participated, which may have contributed to this variability. However, this heterogeneity was intentionally included to reflect clinical routine, where imaging is interpreted by radiologists with different backgrounds.

Despite the moderate agreement, several differences in image quality between reconstruction settings reached statistical significance, indicating that the observed trends were sufficiently robust across readers.

Notably, interobserver variability was particularly high in the evaluation of intrahepatic vessel dichotomization, a feature we had considered especially relevant for pretherapeutic planning. Unfortunately, due to the inconsistent ratings, this question remains unanswered by our current study and warrants further investigation in future work.

Since the arterial phase is the most important phase for HCC imaging, we focused our research on that particular contrast phase. From this experience, we do not expect any improved results for various slice thickness/kernel combinations for the portal venous and delayed phases, as the largest benefits were observed for vessels and for the assessment of structures that are hyperdense in the arterial phase, like the renal cortex.

## 5. Conclusions

In conclusion, this study underscores the importance of optimal protocol choice with first-generation clinically approved PCD-CT scanners. Reconstructions using sharper kernels of Br44 and Br48 at 1 mm slice thickness might be best suited for the assessment of the hepatic vasculature, while the Br40_1 mm_ had the best overall performance for the assessment of the abdominal parenchyma and HCC.

## Figures and Tables

**Table 1:** Acquisition and reconstruction parameters.

Scan Parameters	
Collimation	144 × 0.4 mm
Rotation time	0.5 s
Pitch factor	0.80
Tube potential	120 kVp
CARE keV IQ Level	170
Reconstruction Parameters	
Slice thickness	3 mm/1 mm/0.4 mm
Reconstruction Increment	2 mm/0.8 mm/0.3 mm
Matrix Size	512 × 512
Kernel	Br40/Br44/Br48/Br56
Quantum iterative reconstruction	Level 3
Window	User specific

**Table 2:** Contrast-to-noise ratio and noise of each reconstruction set. CNR, contrast-to-noise ratio; HCC, hepatocellular carcinoma; CHA, common hepatic artery; kidney refers to renal cortex. * *p* < 0.05, ** *p* < 0.01, *** *p* < 0.001 vs. reference standard (Br40–3 mm); missing asterisks refer to *p* > 0.05.

	CNR				Noise
Reconstruction	HCC	Aorta	CHA	Kidney	
Br40–3 mm	4.5 ± 2.0	39.1 ± 13.0	37.9 ± 11.9	15.1 ± 5.2	12.4 ± 1.6
Br44–3 mm	3.5 ± 1.5	30.5 ± 9.6 **	29.8 ± 9.1 *	11.8 ± 4.0 *	15.8 ± 1.9
Br48–3 mm	2.7 ± 1.2 ***	24.2 ± 7.6 ***	23.0 ± 7.3 ***	9.2 ± 3.2 ***	19.8 ± 2.3 ***
Br56–3 mm	2.0 ± 0.8 ***	17.9 ± 5.1 ***	16.8 ± 4.9 ***	6.6 ± 2.3 ***	26.8 ± 2.8 **
Br40–1 mm	3.0 ± 1.2 **	26.5 ± 9.2 ***	27.8 ± 9.7 ***	10.4 ± 3.7 ***	18.4 ± 2.7 ***
Br44–1 mm	2.4 ± 0.9 ***	20.4 ± 6.6 ***	21.4 ± 7.1 ***	8.0 ± 2.7 ***	23.7 ± 3.1 ***
Br48–1 mm	1.8 ± 0.7 ***	15.9 ± 5.2 ***	16.2 ± 5.5 ***	6.1 ± 2.2 ***	30.2 ± 4.2 ***
Br56–1 mm	1.3 ± 0.5 ***	11.4 ± 3.3 ***	11.5 ± 3.6 ***	4.3 ± 1.5 ***	42.1 ± 4.8 ***
Br40–0.4 mm	2.0 ± 0.7 ***	17.6 ± 6.0 ***	18.5 ± 6.6 ***	6.9 ± 2.3 ***	27.7 ± 3.8 ***
Br44–0.4 mm	1.5 ± 0.6 ***	13.3 ± 4.5 ***	14.0 ± 5.0 ***	5.2 ± 1.8 ***	36.6 ± 6.8 ***
Br48–0.4 mm	1.2 ± 0.5 ***	10.5 ± 3.4 ***	10.7 ± 3.7 ***	4.1 ± 1.4 ***	45.6 ± 5.5 ***
Br56–0.4 mm	0.9 ± 0.3 ***	7.6 ± 2.2 ***	7.6 ± 2.5 ***	2.9 ± 1.0 ***	63.8 ± 7.3 ***

## Data Availability

Non-identifiable data will be made available upon reasonable request.
